# Estimation of Tool Wear and Surface Roughness Development Using Deep Learning and Sensors Fusion

**DOI:** 10.3390/s21165338

**Published:** 2021-08-07

**Authors:** Pao-Ming Huang, Ching-Hung Lee

**Affiliations:** 1 Department of Mechanical Engineering, National Chung Hsing University, Taichung 402, Taiwan; g107061308@mail.nchu.edu.tw; 2 Department of Electrical and Computer Engineering, National Yang Ming Chiao Tung University, Hsinchu City 300, Taiwan; 3 Department of Electrical and Computer Engineering, National Chiao Tung University, Hsinchu City 300, Taiwan

**Keywords:** deep learning, vibration, sound, fusion, tool wear, surface roughness, convolution neural network

## Abstract

This paper proposes an estimation approach for tool wear and surface roughness using deep learning and sensor fusion. The one-dimensional convolutional neural network (1D-CNN) is utilized as the estimation model with X- and Y-coordinate vibration signals and sound signal fusion using sensor influence analysis. First, machining experiments with computer numerical control (CNC) parameters are designed using a uniform experimental design (UED) method to guarantee the variety of collected data. The vibration, sound, and spindle current signals are collected and labeled according to the machining parameters. To speed up the degree of tool wear, an accelerated experiment is designed, and the corresponding tool wear and surface roughness are measured. An influential sensor selection analysis is proposed to preserve the estimation accuracy and to minimize the number of sensors. After sensor selection analysis, the sensor signals with better estimation capability are selected and combined using the sensor fusion method. The proposed estimation system combined with sensor selection analysis performs well in terms of accuracy and computational effort. Finally, the proposed approach is applied for on-line monitoring of tool wear with an alarm, which demonstrates the effectiveness of our approach.

## 1. Introduction

In the manufacturing industry, tool wear and surface roughness play important roles during the machining process. Surface roughness and tool wear are performance indices for customers in finished products that affect the machining cost. Therefore, estimation models for tool wear and surface roughness should be developed. Recently, various estimation approaches have been proposed, e.g., artificial neural networks [1,2,3,4,5,6,7,8,9,10], regression [9,11,12,13,14,15,16], support vector machines [17,18,19,20], response surface methodology [21,22], random forest [18,23,24], and adaptive network-based fuzzy inference systems [25,26,27]. In general, the chosen model and data directly affect estimation accuracy. Most of these studies utilized vibration signals and focused on machine learning model selection; variation of the machining conditions was less common. In addition, the effective machining parameters or features should be analyzed and discussed prior to model construction. A variety of data corresponding to the machining parameters should be considered in the data collection process. This results in the effectiveness and performance of the estimation model. In this study, machining experiments with CNC parameters are designed using a uniform experimental design method (UED) to guarantee a variety of collected data.

With the development of artificial intelligence technology, deep learning is a branch of machine learning that can automatically extract features to represent the characteristics of the data [28,29,30,31,32,33]. In this study, we estimate the tool wear and surface roughness using a one-dimensional (1D) convolutional neural network (1D-CNN) with sensor fusion. The machining vibration signals and sound signals were utilized for the estimation. First, correlation analysis according to the Pearson correlation coefficient was used to evaluate the correlation between CNC parameters and tool wear. Subsequently, experiments were designed with different CNC parameter combinations using a uniform experimental design for data collection. During the machining process, vibration signals in three coordinates, sound signals, and spindle current signals were simultaneously collected. Then, an influential sensor analysis was presented to select sensors according to the RMSE of the 1D-CNN. Finally, a 1D-CNN with sensor fusion (X and Y coordinate vibrational signals and sound signals) technique is proposed.

The remainder of this paper is organized as follows. Section 2 introduces tool wear and surface roughness definitions, experimental setups, and data collection. Then, the estimation of tool wear and surface roughness using a 1D-CNN and sensor fusion are presented in Section 3, and Section 4 presents the experimental results and discussion. Finally, Section 5 presents the conclusions.

## 2. Problem Formulation and Experiment Setups

This section introduces the definitions of tool wear and surface roughness, experimental setups for machining, and data collection processes. To accelerate the tool wear status and collect effective data (including labeling), the proposed approach is also presented.

### 2.1. Tool Wear and Surface Roughness

Tool wear is the result of interactions between the physical and chemical domains and is caused by temperature and friction generated during the machining process [34]. According to ISO-8688 [35], tool wear is classified as uniform flank wear, non-uniform flank wear, and localized flank wear. Here, the standard for tool life end points adopts localized flank wear that achieves 0.35 mm to enable early warning. The measurement of tool wear was performed visually. In addition, the surface roughness is a measure of surface texture. A surface profile that results from the intersection of a real surface with a specified plane is quantified by the vertical deviations of a real surface from its ideal form.

### 2.2. Data Acquisition Devices and Measurement of Wear and Roughness

**Figure 1:**
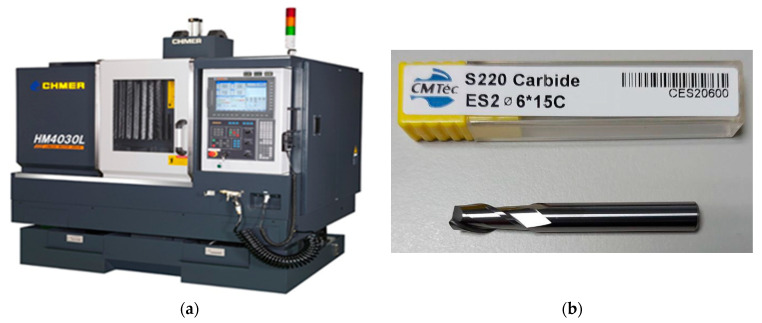
Experiment setup. (**a**) Three-axis CNC machine tool (HM4030L). (**b**) Tungsten carbide end milling cutters with 2 blades and no coating.

**Figure 2:**
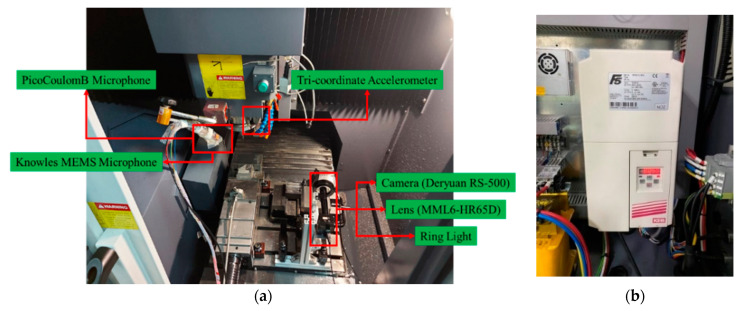
Data acquisition devices. (**a**) The locations of sensors installation. (**b**) Inverter for measuring spindle current.

The experiment was conducted on a three-axis CNC machine tool (CHMER HM4030L), as shown in Figure 1. Figure 1a shows the CNC machine tool with the GENTEC controller. The workpiece material carbon steel (S50C) with low hardness (less than 5 HRC) was selected. The selected cutting tool is tungsten carbide end milling cutters with two blades and no coating, as shown in Figure 1b. In this study, four types of sensors were used, and the mounting locations are shown in Figure 2. Figure 2a shows the installed locations of the three-coordinate accelerometer and two sound sensors, and the current signals of the spindle are collected by the inverter (shown in Figure 2b). The vibration signals were collected from a tri-coordinate accelerometer (CTC AC230) that was mounted beside the spindle. The sound signals were collected from the PCB microphone and the Knowles MEMS microphone, which were fixed on a magnetic base holder. The current of the spindle was collected by inverters (KEB Combivert).

**Figure 3:**
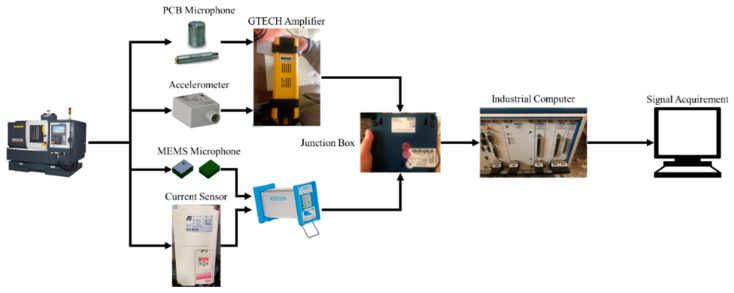
Illustration of signal acquisition.

Figure 3 illustrates the flow chart for signal collection, which is summarized as follows. The sensor MEMS microphone, PCB microphone, tri-coordinate accelerometer, and inverter are installed as described above. Subsequently, the preprocessing operations, power amplification, and filtering were done for the collected signals. Herein, all signals were terminated at the junction box and an industrial computer with a capture module was used to acquire signals. The NI PXIe-6361 multifunction I/O module with a sampling rate of 100 kHz was applied because of the maximum sampling rate of these sensors, which is 20 kHz, and the NI PXIe-6361 multifunction I/O module with a sampling rate of 100 kHz was applied. To obtain the signals, LabView was used to build an environment for signal observation and to acquire signals.

**Figure 4:**
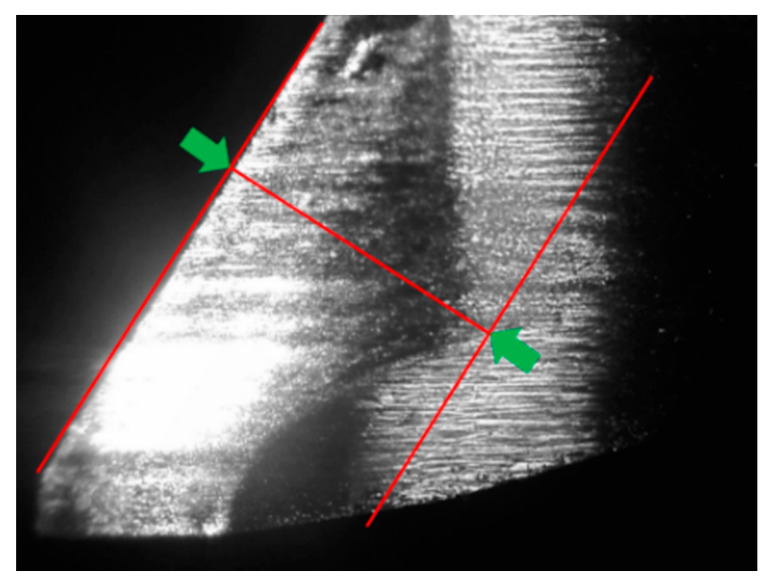
Calculation of the distance between the unworn contour and the location of maximum wear.

For tool wear measurement, a camera (Deryuan RS-500), lens (MML6-HR65D), and ring light were mounted inside the machine tool, as shown in Figure 2a. The public image processing software ImageJ [36] was used to calculate the distance between the unworn contour and the location of maximum wear, as shown in Figure 4. For surface roughness measurements, the Surftest SJ400 (Mitutoyo) was used to measure the surface roughness.

### 2.3. Signal Analysis and Processing

**Figure 5:**
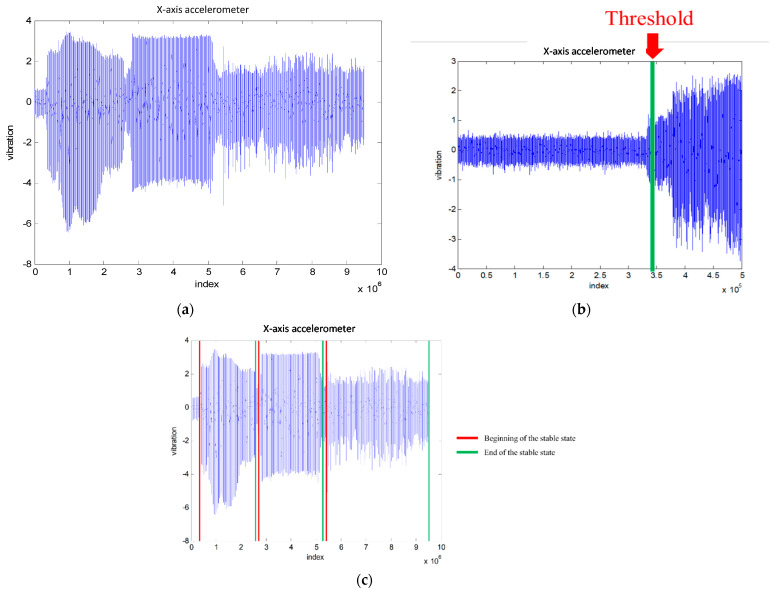
Signal analysis and processing, (**a**) The original signal data from X-coordinate accelerometer. (**b**) The threshold of the stable state. (**c**) The stable state.

The machining processing is defined as the time between the moment that the cutting tool touches the workpiece to the moment that the tool leaves the workpiece completely. However, there exist unstable signals, which might be caused by the impact of the cutting tool on the workpiece or a situation in which the spindle of machine tool has not warmed up. Thus, the collected signal should be preprocessed to remove it. In this paper, the stable status is defined as twice the magnitude of the signal when not machining as the threshold to decide the beginning of the stable state and end of the stable state. Figure 5a–c show that process of the signal cutting illustrated by the X-axis vibrational signals; Figure 5a is the original data; The threshold of the stable state is shown in Figure 5b; and Figure 5c shows the stable state signals after preprocessing. In general, normalization is the important procedure to reduce the variability of each cutting tool based on the various characteristics of cutting tools. Herein, we compute the root mean square (RMS) of the data over 1 s for normalization.

## 3. Estimation Model Development Using CNN and Sensor Fusion

**Figure 6:**
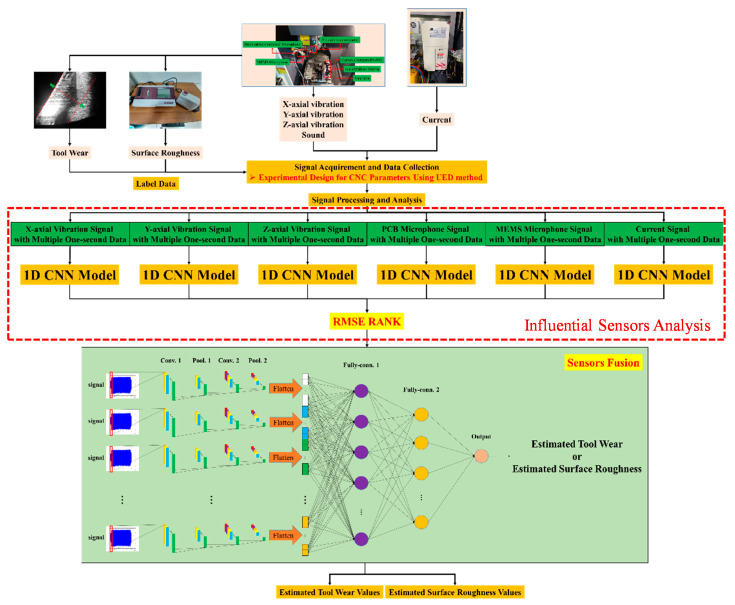
Flow chart of the proposed methodology.

This section introduces the estimation of tool wear and surface roughness using deep learning and sensor fusion. The flowchart of the proposed approach is shown in Figure 6. First, a correlation analysis between CNC parameters and performance indices (tool wear and surface roughness) is introduced for parameter selection. Subsequently, a UED is adopted to design experiments for machining and data collection [37]. Simultaneously, signals are collected from the sensors, and the corresponding tool wear and surface roughness are measured. All signals are sampled at 100k Hz and the sampling duration is 1 second, i.e., the data length is 10^5^. After data pre-processing, an influential sensor analysis is introduced to minimize the number of sensors with accuracy preservation. A 1D-CNN with raw data inputs is adopted to develop the estimation model. Finally, the tool wear is estimated and the surface roughness system is developed.

### 3.1. One-Dimensional Convolutional Neural Network (1D-CNN)

**Figure 7:**
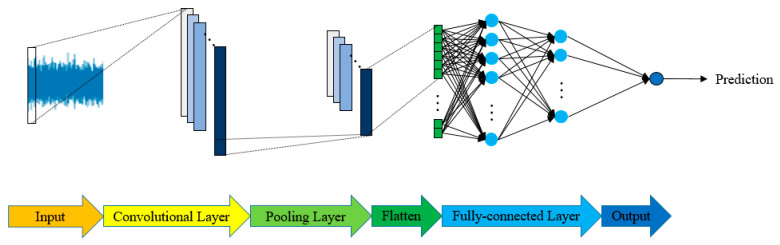
The structure of 1D-CNN.

Deep learning is a branch of machine learning [29,30,38]. By linear or non-linear transforms in multiple hidden layers, it is hoped that features that are sufficient to represent the characteristics of the data can be automatically extracted. In traditional machine learning, features are usually generated from an algorithm that is programmed by humans. The effective features are generated by experts conducting many analyses and discussions from data and understanding the characteristics of the data. It is also called feature engineering. Deep learning has the ability to automatically extract features (feature extraction) and can be regarded as feature learning, which can save time spent on feature engineering. The most common deep learning method is convolutional neural networks (CNNs), proposed by prof. LeCun [38]. In many research fields, CNNs are applied for the classification and prediction of signals and images. Feature extraction can be conducted automatically by convolution operations [39,40]. Recently, this process has been utilized for signal classification and prediction [30,31,32,38]. It has the ability to carry out input signal convolutional operations in the local area to get one-dimensional features, and various kernels extract specific characteristics from the input signals. This means that the CNN can be developed for automatic feature learning instead of feature extraction and selection by human experts. The inputs of 1D-CNN can be raw signal data or other one-dimensional data. The typical structure of a CNN is composed of three parts, which are the convolutional layers, pooling layers, and fully connected layers. CNNs make use of filters (also known as kernels) to automatically detect proper features. We here briefly introduce the utilized 1D-CNN, as shown in Figure 7, as follows.

Input: The input of 1D-CNN is one-dimensional data, for example, vibration signals.

Convolution layer: It applies the convolution operation to input and pass the result to the next layer. After convolution of inputs and filters, the feature map is obtained from activation function mapping. The filter operation and feature map of the kth convolutional layer is:

(1) $$z_{l}^{k}=f\left(\alpha_{l}*x+b\right)$$

(2) $$z^{k}=\left[z_{1}^{k},\;z_{2}^{k},\;\ldots ,\;z_{N}^{k}\right]$$

(3) $$\mathrm{Length}\;L_{z}=\mathrm{ceil}\left(\frac{L-L_{C}}{S_{CL}}\right)$$

where $x\in {ℛ}^{1\times L}$ is the input, N is the filter number, l is index of filters (l=1, 2, …, N), k is the index of the convolutional layer, $z_{l}^{k}$ is the feature map, f is the non-linear activation function, $\alpha_{l}$ is the kernel matrix of lth filter, b is the bias, L represents length of inputs, $L_{C}$ represents length of filters in convolutional layers, and $S_{CL}$ represents strides of length in convolutional layers.

Pooling Layer: Pooling layers play the role of retaining the information of feature maps. The command pooling method is max-pooling, as shown in following equation.

(4) $$p_{l}^{k}=max\left(z_{j}^{k},\;z_{j+1}^{k},\;\ldots ,\;z_{j+L_{P}}^{k}\right)$$

(5) $$p^{k}=\left[p_{1}^{k},\;p_{2}^{k},\;\ldots ,\;p_{\mathrm{ceil}\left(\frac{L_{z}}{L_{P}}\right)}^{k}\right]$$

where r is the index of features after pooling where $r=1,\;2,\ldots ,\;\mathrm{ceil}\left(\frac{L_{z}}{L_{P}}\right)$, $L_{P}$ represents the length of filters in pooling layers, and $p_{l}^{k}$ is the pooling result of the lth feature map after convolving the kth convolutional layer.

Flatten: The feature maps that are the result of convolution and pooling are flattened into one-dimensional features. The equation is shown below:

(6) $$y=f\left(\sum_{a=1}^{n}w_{a}h_{a}+b\right)$$

where $h_{a}$ is the input of the neuron, $w_{a}$ is the weight of input $h_{a}$, a=1, 2, …, n, *b* is the bias, *f* is the activation function of the neuron, and *y* is the output of the neuron.

Fully connected layer: The fully connected layer is a backpropagation neural network. Herein, the estimation problem is applied, thus, the linear activation function is adopted.

### 3.2. Parameters Analysis

**Figure 8:**
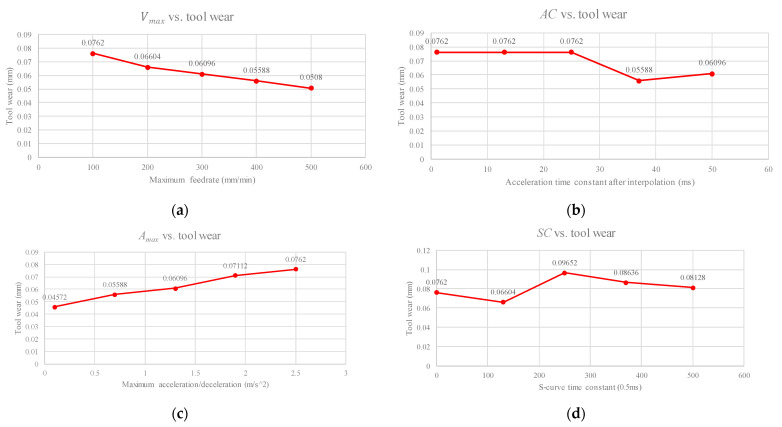
Results of correlation analysis—CNC parameters versus tool wear. (**a**) *V_max_*; (**b**) *AC*; (**c**) *A_max_*; and (**d**) *SC*.

First, an experiment analysis is presented to select the machining parameters, and the corresponding machining path with a length of 100 mm, a cutting width of 2 mm, and a cutting depth of 1.5 mm is designed. There are many machining parameters in the CNC controller; here, we selected four general parameters (federate, acceleration time constant, maximum acceleration, and S-curve time constant) according to the interpolation method [10,41], and the corresponding ranges are shown in Table 1. Figure 8 shows the experimental design and results for the relationship between the CNC parameters, *V_max_*, *AC*, *A_max_*, *SC*, and tool wear. The corresponding Pearson product-moment correlation coefficient computation (PCC) values are −0.9864, −0.4674, 0.9934, and 0.4162, respectively. From the values of PCC, the maximum feed rate ($V_{max}$) was negatively correlated with tool wear. The maximum acceleration/deceleration ($A_{max}$) was positively correlated with tool wear. The acceleration time constant after interpolation (AC) and the *S*-curve time constant (*SC*) are less correlated with tool wear. According to the experimental results, AC and *SC*, which are related to the interpolation computation of the controller, have less influence on tool wear. However, $V_{max}$ and $A_{max}$, which are related to the cutting theory, have a greater influence on tool wear. Therefore, $V_{max}$ and $A_{max}$ were selected for the design of machining experiments using a UED [36]. Table 2 and Table 3 show the CNC parameter levels and parameter combinations based on the UED. The selection of CNC parameter levels was based on the suggestion of the cutler. Then, because of the finished cut, which requires a slow feed rate to smoothen the surface of the material, we set the maximum level of $V_{max}$ to 360 mm/min.

**Figure 9:**
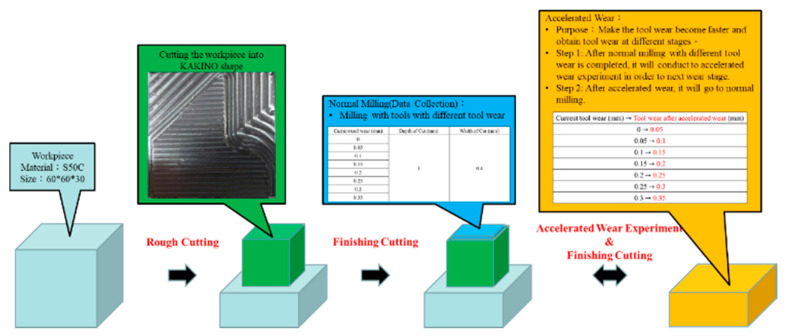
Illustration of experiments for tool wear.

The experimental design is presented in Table 4 and Figure 9. Figure 9 shows a schematic of the experiment. In this study, two modes of the machining method were created in the experiment, namely normal milling and accelerated wear. Accelerated milling is designed to speed up the tool wear and to obtain the tool wear at different stages. In order to be closer to the actual industrial machining, three levels of $V_{max}$ and $A_{max}$ were selected in normal milling. The following procedure describes the experimental operations.

Experiment Procedure:

Step 1. Experimental Design—CNC parameters are selected based on an analysis of the CNC parameter and tool wear, and the combination of parameters is determined using the UED method (Table 3).

Step 2. Rough Cutting—Rough cutting is carried out to obtain the workpieces with a KAKINO shape.

Step 3. Finished Cutting and Data Collection—The finished cutting is employed to acquire the signals. When one finished cutting is completed, the cutting tool needs to be replaced, and the signal from these sensors must be saved.

Step 4. Measurement and Label—The tool wear value is measured using a camera (Deryuan RS-500) with the image processing tool (ImageJ), and the surface roughness is measured usin Surftest SJ400 (Mitutoyo).

Step 5. Accelerated wear—After one finished cutting is completed, the accelerated wear experiment is conducted to obtain the next tool wear stage.

Step 6. Repeat Steps 4–6 until the tool wear stage reaches 0.35 mm.

### 3.3. Influential Analysis for Sensor Selection

In industrial applications, low-cost and minimum add-on sensors are preferred for manufacturing. We here present an influential analysis pertaining to sensor selection. As mentioned above, even if some sensors are mounted on machine tools for research applications, there are also differences between these sensors, which are suitable for various applications. Signals that are collected by unsuitable sensors may cause the model recognition or prediction to become poor. This causes a difference in the results of classification or estimation based on the signals from these sensors as training data for the model. Therefore, it is important to select an appropriate sensor for model training.

Based on the concept of [42], sensors whose estimation is better using 1D-CNN are chosen, where the model input is the signal and the model output is the tool wear or surface roughness. The procedure is as follows:

Procedure for Sensor Selection

Step 1: Sensor selection—All signals of the six sensors are obtained to investigate the tool wear and surface roughness.

Step 2: Construct the 1D-CNN model—Six 1D-CNN models are established using each sensor’s raw signal as the model input and tool wear or the surface roughness as the model output. Figure 7 shows the construction of the 1D-CNN model for tool wear and surface roughness estimation.

Step 3: Calculate the RMSE values of each prediction model, which corresponds to the prediction error of the model.

Step 4: Ranking by RMSE—Sort the RMSE values of each tool wear and surface roughness estimation model.

Step 5: Influential sensor selection—Select the sensor signals according to the RMSE ranking.

Step 6: Sensor number decision—Determine the number of sensors according to requirements and cost.

Finally, the raw data of the selected sensors are the inputs of the proposed monitoring system, as shown in Figure 6. In actual machining, many uncertain factors affect the results of the sensors. The use of small sensors for collecting data or monitoring states is more influenced by external factors. In addition, it is impossible to use a few small sensors to obtain all the information of the machining condition. By combining the characteristics of sensors, external noises can be reduced. Therefore, sensor fusion plays an important role in the acquisition of data or the monitoring of states in the machining process.

## 4. Experimental Results and Discussions

In this section, the experimental results of tool wear and surface roughness are presented, and the relationship between tool wear and surface roughness is discussed. Subsequently, the relevant sensor selection is discussed. Finally, the 1D-CNN combined with sensor fusion based on the relevant sensor selection analysis is introduced, and the experimental results are presented.

### 4.1. Relationship between Tool Wear and Surface Roughness

**Figure 10:**
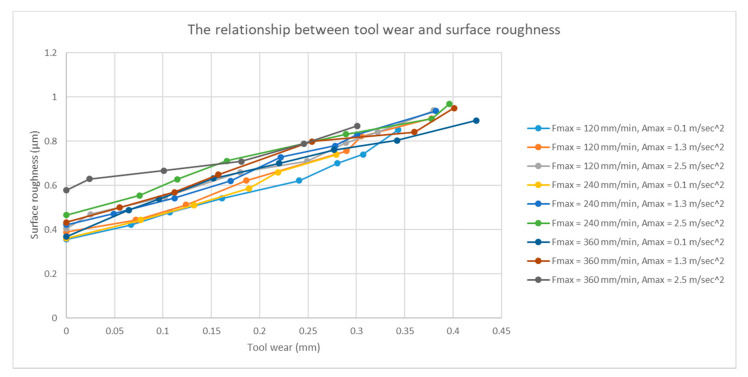
Relationship between tool wear and surface roughness under different machining conditions.

Surface roughness is an important indicator that is used to measure the quality of finished products. The quality of the cutting tool influences the surface roughness of the workpiece when the cutting tool contacts the workpiece during the machining process. Thus, tool wear, which is a process of continuous variation, affects surface roughness. Therefore, the relationship between tool wear and surface roughness should be verified. We used the KAKINO path for actual milling. The relationship between tool wear and surface roughness is shown in Figure 10. From the experimental results and the PCC computation, it can be observed that tool wear is positively related to the surface roughness. Table 5 lists the PCC values of the different experiments. It can be verified that tool wear and surface roughness are positively related.

### 4.2. Results of Influential Sensor Selection Analysis

**Figure 11:**
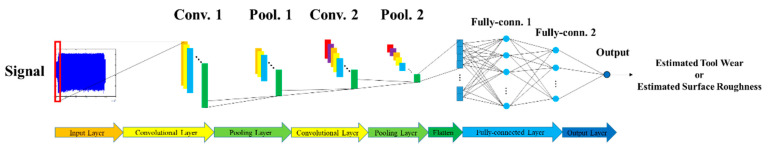
Structure of 1D-CNN.

As mentioned above, the 1D-CNN model is established for the estimation of tool wear or surface roughness. The utilized 1D-CNN structure is shown in Figure 11 and Table 6. Here, six sensors are labeled by Sensors 1–3: X-Y-Z coordinate accelerometer (vibration signals), Sensor 4: PCB microphone (sound signals), Sensor 5: Knowles MEMS microphone (sound signals), and Sensor 6: spindle current signals. Table 7 introduces the data for different machining conditions. The sample length is 100,000 after the signal analysis and processing introduced in Section 2. Note that the data numbers are not the same due to different machining conditions. The training, validation, and testing data were 70%, 15%, and 15% of the data, respectively. In addition, the corresponding hyper-parameters for the influential sensor selection analysis are shown in Table 8. Table 9 and Table 10 show the corresponding estimated RMSE values of the testing data of the tool wear and surface roughness using 1D-CNN under different machining conditions and sensors, respectively. The definition of RMSE is as follows:

(7) $$\mathrm{RMSE}=\sqrt{\frac{1}{n}\sum_{i=1}^{n}{\left(y_{i}-{\hat{y}}_{i}\right)}^{2}}$$

where $y_{i}$ is the predicted value, ${\hat{y}}_{i}$ is the actual value, and n is the number of data points. It can be found that the average predictive ability of the tri-coordinate accelerometers in the estimation of tool wear are improved, and the estimation of the surface roughness of the tri-coordinate accelerometers is similar to the average estimation ability of the PCB microphone and the Knowles MEMS microphone. According to the results shown in Table 8 and Table 9, we can conclude that the relative influence of sensors for tool wear is sensor 2 > sensor 1 > sensor 3 > sensor 4 > sensor 5 > sensor 6, that is, influence ranking as Y-coordinate, X-coordinate, Z-coordinate, PCB microphone, Knowles MEMS microphone, and spindle current, respectively. In addition, for the surface roughness, the corresponding ranking is Y-coordinate, X-coordinate, PCB microphone, Knowles MEMS microphone, Z-coordinate, and spindle current, respectively. The results were employed for sensor fusion using 1D-CNN.

### 4.3. Estimation of Tool Wear and Surface Roughness Using 1D-CNN with Sensors Fusion

Based on the above results of the sensors’ influential ranking, we designed an experimental validation process by adding sensor signals step-by-step from the influence ranking. Table 11 shows the RMSE values of the testing data for the tool wear and the surface roughness for different sensor fusions. Note that ‘Number’ denotes the number of sensors for the CNN input, for example, if Number is equal to three, the inputs of the model are the X -, Y -, and Z-coordinates for estimation. Based on the experimental results obtained, the best RMSE of the testing data is a CNN that uses five sensor inputs. However, the corresponding RMSE values that were computed by the five models using these five signals as input data were very close. This means that the five sensors have a good predictive effect on the tool wear and surface roughness. Therefore, the best sensors for model training can be filtered out using the prediction results of a single sensor.

However, in the actual manufacturing of industrial applications, it is impossible to mount a large number of sensors on machine tools during the machining process because of cost considerations, machining environment constraints, and the ease of sensor installation. In this study, according to the design of the experimental path, the KAKINO path is a two-dimensional (2D) plane (X-Y plane). Therefore, based on the cutting theory, the signals of the X-coordinate and Y-coordinate accelerometers should be considered. To reduce the cost, Knowles MEMS microphone is an industrial technology that combines microelectronics and mechanical engineering, and it is cheaper than other sensors that are sound acquisition devices. Thus, the experiment is designed to combine the signals of the X-coordinate, Y-coordinate, and Knowles MEMS microphone as inputs of the 1D-CNN using the sensor fusion method. The corresponding RMSE values of the testing data of the tool wear and surface roughness were 0.0566 mm and 0.0592 μm, respectively. The results show that the estimation performance obtained, considering the cost reduction, can yield better results.

### 4.4. Verification of Influential Sensor Selection Analysis

**Figure 12:**
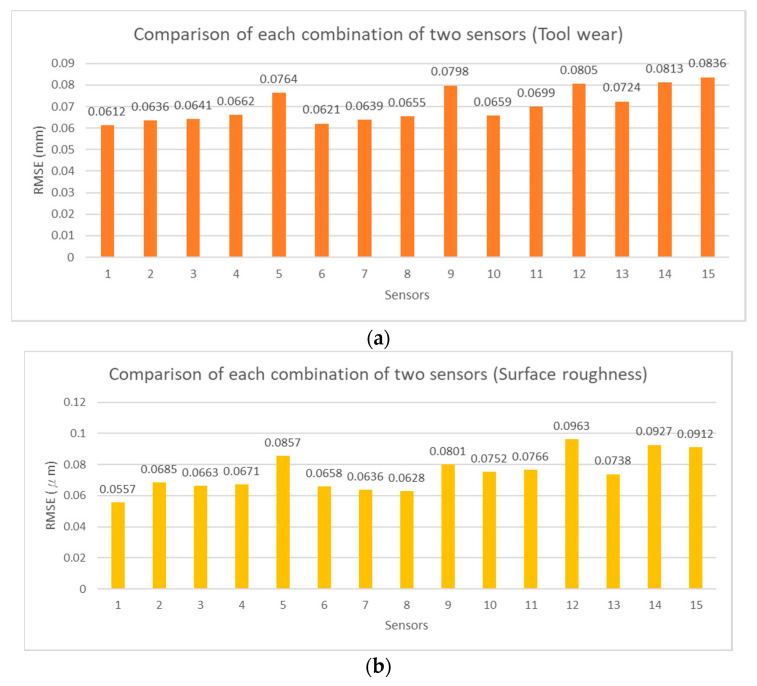
Comparison of each combination of two sensors, (**a**) tool wear; (**b**) surface roughness.

**Figure 13:**
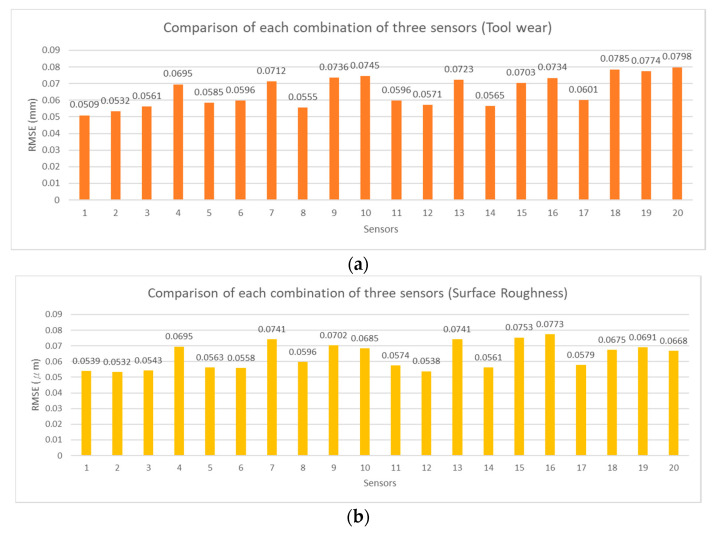
Comparison of each combination of three sensors, (**a**) tool wear; (**b**) surface roughness.

**Figure 14:**
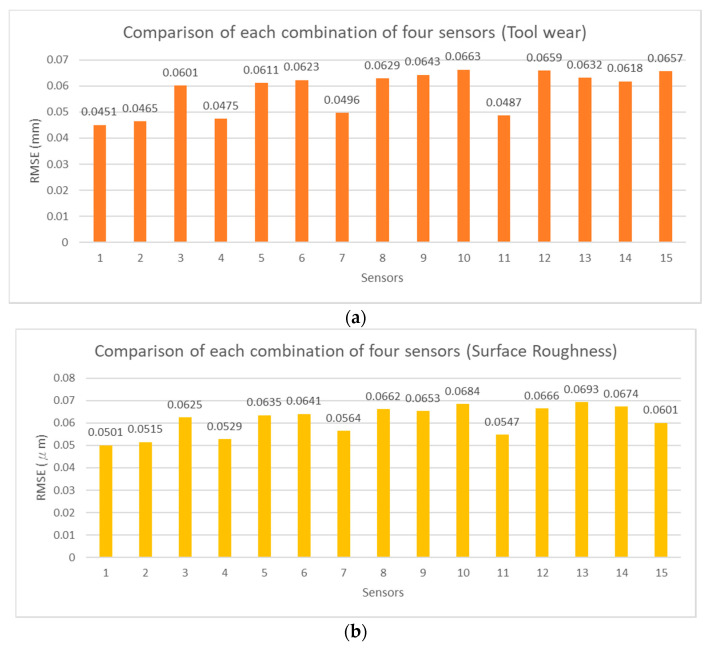
Comparison of each combination of four sensors, (**a**) tool wear; (**b**) surface roughness.

**Figure 15:**
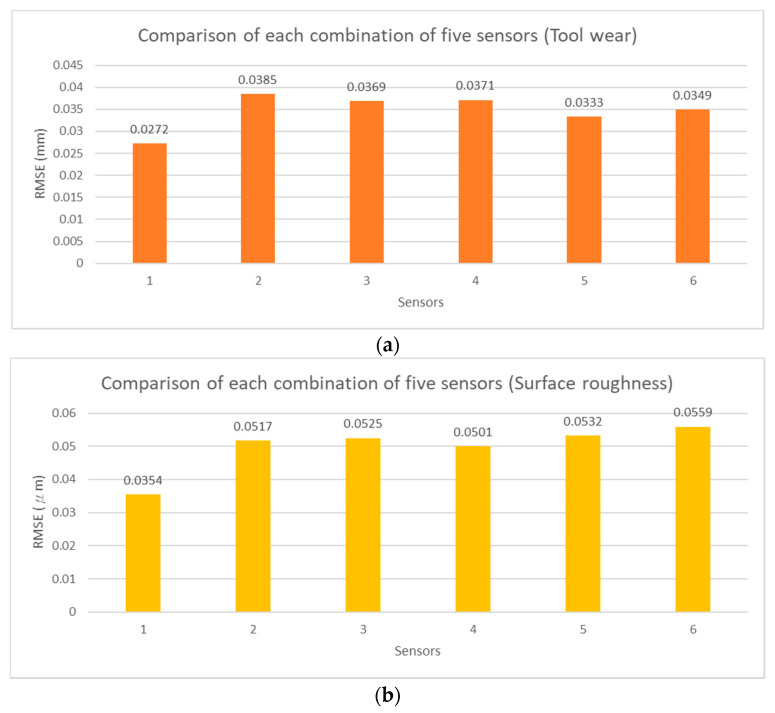
Comparison of each combination of five sensors, (**a**) tool wear; (**b**) surface roughness.

To verify the feasibility of the influential sensor selection analysis, the exhaustion method was adopted to estimate tool wear and surface roughness with different numbers of sensor combinations. The RMSE values of each estimation model are shown in Figure 12, Figure 13, Figure 14 and Figure 15 for two, three, four, and five sensors, respectively. The current signals are removed owing to the incorrect RMSE results in Table 11. Figure 12, Figure 13, Figure 14 and Figure 15 show the predicted RMSE results of the tool wear and surface roughness in (a) and (b), respectively. According to the experimental results, the best estimation performances of different sensor combinations are generally consistent with the results of the influential sensor selection analysis. Table 12 shows a comparison of the results of the method of exhaustion and influential sensor selection analysis for tool wear and surface roughness, respectively. The results of the exhaustion method are the same as those of the influential sensor analysis. This validation demonstrates the effectiveness of the proposed approach, which has the ability to select the sensors.

### 4.5. Demonstration of On-Line Monitoring

**Figure 16:**
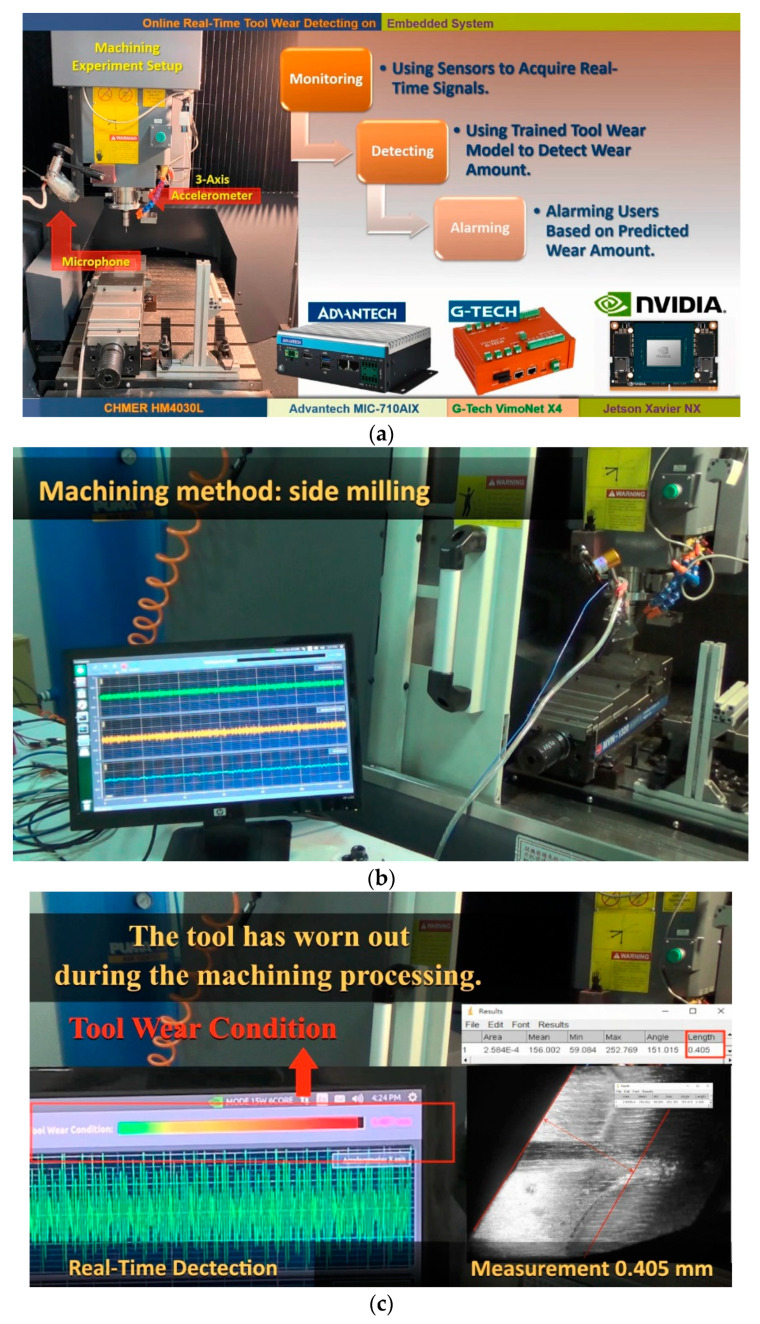
On-line monitor implementation. (**a**) Hardware architecture; (**b**) machining method illustration; and (**c**) monitoring result with alarm and validation (measured off-line).

Herein, the on-line monitoring result is introduced to demonstrate the effectiveness of the proposed approach. As result of sensor selection, X- and Y-acceleration vibration signals and sound signals are adopted to implement in the on-line monitoring. The proposed system acquires real-time signals by sensors and detects the tool wear amount by the trained AI model. Finally, alarming users based on the predicted wear amount. The Advantech industrial PC with CPU (ARMv8.2 HMP CPU) and NVIDIA GPU (384 CUDA Cores, 48 Tensor Cores, Performance up to 21 TOPS) is utilized, as illustrated in Figure 16. Figure 16a introduces hardware architecture and monitoring; Figure 16b shows the machining method; and Figure 16c is the monitoring result with alarm and validation (measured off-line). As shown in Figure 16b, three signals are real-time acquired and shown, and visual monitoring using a color bar is used for the alarm (shown in Figure 16c). The results demonstrate the effectiveness of our proposed approach.

## 5. Conclusions

This paper introduced a tool wear and surface roughness estimation system using deep learning and sensor fusion techniques. A one-dimensional convolutional neural network (1D-CNN) with X-and Y-coordinate vibration signals and sound signal fusion was proposed. The machining experiments with CNC parameters were designed by UED to guarantee a variety of collected data. The vibration, sound, and spindle current signals were collected and labeled according to the machining parameters. In addition, we proposed an influential sensor selection analysis to preserve the estimation accuracy and minimize the number of sensors. To verify the feasibility of influential sensor selection analysis, the method of exhaustion was adopted to estimate tool wear and surface roughness with different numbers of sensor combinations. The results show that the best estimation performances of different sensor combinations are mostly consistent with the results of the influential sensor selection analysis. Finally, the estimation system of tool wear and surface roughness with five sensors has the best estimation performance, the RMSE of tool wear is 0.0286 mm, and the RMSE of the surface roughness is 0.0368 μm. We propose an experiment that combines the signals of the X-, Y-, and Knowles MEMS microphones for cost reduction. The corresponding RMSE of the testing data from the estimated tool wear model was approximately 0.0566 mm, and the RMSE of the testing data from the estimated tool wear model was approximately 0.0592 μm. Finally, the proposed approach has been applied to on-line monitoring of tool wear with an alarm, which demonstrates the effectiveness of our approach.

## Figures and Tables

**Table 1:** Range of CNC parameters.

CNC Parameter	Range	Unit
Maximum feed rate ($V_{max}$)	0–6000	mm/min
Acceleration time constant after interpolation (AC)	1–50	ms
Maximum acceleration ($A_{max}$)	0.001–2.5	m/s^2^
S-curve time constant (SC)	0–500	0.5 ms

**Table 2:** Selection level of UED for *V_max_* and *A_max_*.

Level	$V_{max}$ (mm/min)	$A_{max}$ $\;(m/s^{2})$
Level 1	120	0.1
Level 2	240	1.3
Level 3	360	2.5

**Table 3:** Selected values of *V_max_* and *A_max_*.

Experiment	$V_{max}$ (mm/min)	$A_{max}$ $\;(m/s^{2})$
EX 01	120	0.1
EX 02	120	1.3
EX 03	120	2.5
EX 04	240	0.1
EX 05	240	1.3
EX 06	240	2.5
EX 07	360	0.1
EX 08	360	1.3
EX 09	360	2.5

**Table 4:** Experimental design for machining.

Items	Unit	Normal Milling	Accelerated Wear
Purpose	--	Signal Collection	Accelerated Wear
Workpiece Material	--	S50C	S50C
Material Hardness	HRC	<5	50
$V_{max}$	mm/min	360/240/120	20
$A_{max}$	$m/s^{2}$	0.1/1.3/2.5	0.3
Spindle Speed	rpm	8000	8000
Milling Path	--	KAKINO	Straight
Depth of Cut	mm	1	0.9
Width of Cut	mm	0.4	0.9

**Table 5:** Pearson’s correlation coefficient in different experiments.

Experiment	$V_{max}\;(\mathrm{mm}/\min)$	$A_{max}$ $\;(m/s^{2})$	Pearson’s Correlation Coefficient
EX01	120	0.1	0.9869
EX02	120	1.3	0.9939
EX03	120	2.5	0.9953
EX04	240	0.1	0.9939
EX05	240	1.3	0.9969
EX06	240	2.5	0.9933
EX07	360	0.1	0.9876
EX08	360	1.3	0.9897
Ex09	360	2.5	0.9790
Average			0.9907

**Table 6:** Structure of 1D-CNN for the estimation of tool and surface roughness.

Layers	Filter Size	Stride	Number of Filters or Nodes	Activation Function
Conv. 1	36	5	30	Sigmoid
Pool. 1	20	--		
Conv. 2	18	5	30	Sigmoid
Pool. 2	10	--		
Flatten	--			
Fully connected 1	--		128	Sigmoid
Fully connected 2			64	Sigmoid
Outputs			1	None

**Table 7:** The numbers of data in different machining conditions.

Conditions	Sensors						Total
	Sensor 1	Sensor 2	Sensor 3	Sensor 4	Sensor 5	Sensor 6	
F120A0.1	654	636	562	577	657	362	3448
F120A1.3	548	497	560	429	517	268	2819
F120A2.5	684	562	449	530	533	461	3219
F240A0.1	264	268	239	211	247	184	1413
F240A1.3	357	347	302	228	307	252	1793
F240A2.5	291	305	239	271	291	227	1624
F360A0.1	221	233	197	170	218	160	1199
F360A1.3	211	218	172	147	157	144	1048
F360A2.5	154	139	148	137	151	191	920
Total	3384	3205	2868	2700	3078	2249	--

**Table 8:** Hyperparameters of 1D-CNN for influential sensor selection analysis.

Hyperparameter	Types or Values
Epoch	5000
Batch Size	16
Learning Rate	0.00001
Loss Function	Mean Square Error
Optimizer	Adamax

**Table 9:** RMSE values of testing data for tool wear.

Conditions	RMSE Values of Testing Data (mm)					
	Sensor 1	Sensor 2	Sensor 3	Sensor 4	Sensor 5	Sensor 6
F120A0.1	0.0506	0.0503	0.053	0.0464	0.0438	0.0969
F120A1.3	0.058	0.0627	0.0621	0.0631	0.0696	0.0917
F120A2.5	0.0577	0.0451	0.0465	0.0598	0.0524	0.0918
F240A0.1	0.0596	0.0572	0.0533	0.0781	0.0741	0.0981
F240A1.3	0.074	0.0718	0.0872	0.073	0.0849	0.1022
F240A2.5	0.1116	0.0959	0.109	0.106	0.1279	0.1212
F360A0.1	0.0994	0.1129	0.1232	0.1395	0.1413	0.1276
F360A1.3	0.0833	0.0809	0.0812	0.0854	0.0886	0.139
F360A2.5	0.1031	0.0977	0.1035	0.1039	0.1051	0.1373
Average	0.0775	0.0749	0.0799	0.0839	0.0875	0.1116

**Table 10:** RMSE values of testing data for surface roughness.

Conditions	RMSE Values of Testing Data (μm)					
	Sensor 1	Sensor 2	Sensor 3	Sensor 4	Sensor 5	Sensor 6
F120A0.1	0.0736	0.0587	0.0863	0.0744	0.0791	0.0941
F120A1.3	0.0737	0.0741	0.072	0.0906	0.0993	0.1049
F120A2.5	0.0683	0.0488	0.057	0.0706	0.0704	0.1061
F240A0.1	0.0722	0.0663	0.0635	0.0758	0.0851	0.1246
F240A1.3	0.1042	0.0964	0.1237	0.1126	0.1149	0.1239
F240A2.5	0.1158	0.1239	0.1285	0.1016	0.1048	0.1312
F360A0.1	0.1139	0.1132	0.1256	0.1134	0.1208	0.1329
F360A1.3	0.1134	0.1035	0.1302	0.1019	0.1095	0.1328
F360A2.5	0.1025	0.1015	0.1058	0.1026	0.1086	0.1273
Average	0.0931	0.0874	0.0992	0.0937	0.0992	0.1197

**Table 11:** RMSE values of testing data of the tool wear for different sensor fusions.

**Tool Wear**						
**Number**	**Sensor 2**	**Sensor 1**	**Sensor 3**	**Sensor 4**	**Sensor 5**	**Sensor 6**
1	0.0977 mm	--				
2	0.0626 mm		--			
3	0.0523mm			--		
4	0.0465 mm				--	
5	0.0286 mm					--
6	0.0529 mm					
**Surface Roughness**						
**Number**	**Sensor 2**	**Sensor 1**	**Sensor 4**	**Sensor 5**	**Sensor 3**	**Sensor 6**
1	0.1015 μm	--				
2	0.0571 μm		--			
3	0.0546 μm			--		
4	0.0543 μm				--	
5	0.0368 μm					--
6	0.0653 μm					

**Table 12:** Comparison of influential sensor selection analysis and method of exhaustion.

Number of Sensors	Tool Wear	
	Influential Sensor Selection Analysis	Method of Exhaustion
1	Sensor 2	Sensor 2
2	Sensors 1 and 2	Sensors 1 and 2
3	Sensors 1, 2, and 3	Sensors 1, 2, and 3
4	Sensors 1, 2, 3, and 4	Sensors 1, 2, 3, and 4
5	Sensors 1, 2, 3, 4, and 5	Sensors 1, 2, 3, 4, and 5
**Number of Sensors**	**Surface Roughness**	
1	Sensors 2	Sensors 2
2	Sensors 1 and 2	Sensors 1 and 2
3	Sensors 1, 2, and 3	Sensors 1, 2, and 3
4	Sensors 1, 2, 4, and 5	Sensors 1, 2, 3, and 4
5	Sensors 1, 2, 3, 4, and 5	Sensors 1, 2, 3, 4, and 5
